# Intravitreal Cidofovir Injection for Refractory End‐Stage Glaucoma and Vision Loss in Silicone Oil‐Filled Eyes Following Retinal Reattachment Surgery in Dogs: Four Cases

**DOI:** 10.1111/vop.70185

**Published:** 2026-04-27

**Authors:** Sunhyo Kim, Jaeho Shim

**Affiliations:** ^1^ Ilsan Animal Medical Center Goyang‐si Republic of Korea; ^2^ Department of Veterinary Ophthalmology, College of Veterinary Medicine Gyeongsang National University Jinju‐si Gyeongsangnam‐do Republic of Korea; ^3^ Institute of Animal Medicine, College of Veterinary Medicine Gyeongsang National University Jinju Republic of Korea

**Keywords:** cidofovir, dog, glaucoma, intravitreal injection, retinal reattachment surgery, silicone oil

## Abstract

**Objective:**

To describe the clinical outcome of intravitreal cidofovir injection for palliative management of refractory end‐stage glaucoma and vision loss in silicone oil‐filled eyes following retinal reattachment surgery in dogs.

**Procedures:**

Medical records of dogs that underwent a single intravitreal cidofovir injection for pharmacologic ciliary body ablation were retrospectively reviewed. Data collected included clinical history, intraocular pressure (IOP), post‐injection ocular comfort, complications, and follow‐up duration.

**Results:**

Four dogs (four eyes) were included. All treated eyes demonstrated a marked reduction in IOP at the first post‐injection recheck (14–28 days after injection; post‐injection IOP range: 3–7 mmHg). Adequate pain control was achieved in all dogs throughout the follow‐up period (median follow‐up: 306.5 days; range: 40–369 days). Progressive phthisis bulbi developed in one case at long‐term follow‐up, while the remaining eyes maintained acceptable globe integrity and cosmetic appearance. Moderate to severe post‐injection ocular discharge, conjunctival hyperemia, and aqueous flare were observed in all cases and were manageable with routine topical therapy. All owners reported satisfaction with post‐injection comfort and appearance.

**Conclusions:**

A single intravitreal cidofovir injection may represent a feasible palliative option for controlling intraocular pressure and ocular pain in silicone oil‐filled eyes with refractory glaucoma when enucleation is declined. Further studies are needed to better characterize long‐term safety and outcomes in this clinical setting.

## 1 Introduction

Pharmacologic ciliary body ablation, including intravitreal injection of gentamicin or cidofovir, has been utilized in dogs with chronic, end‐stage glaucoma as a palliative treatment to reduce intraocular pressure (IOP) and alleviate ocular pain [1, 2]. Among these agents, cidofovir has gained popularity due to its favorable safety profile and efficacy after a single administration in most cases [3, 4, 5]. It is believed to exert its effect through selective necrosis of the ciliary epithelium, leading to aqueous humor suppression and sustained hypotony [3, 4, 5]. This technique has become widely accepted, particularly in blind, painful eyes where visual restoration is no longer a therapeutic goal [1, 3, 4].

Retinal reattachment surgery (RRS), typically involving pars plana vitrectomy and endolaser retinopexy, has been associated with several postoperative complications in dogs, including corneal ulceration, cataract formation or progression, silicone oil (SiO) migration into the anterior chamber, and recurrent retinal detachment, among which secondary glaucoma is considered one of the most frequent and clinically significant complications [6, 7, 8, 9, 10]. Chronic IOP elevation in such cases often results in progressive retinal degeneration and optic nerve damage, ultimately leading to irreversible vision loss; although antiglaucoma medications may offer temporary relief, many of these eyes eventually require enucleation, intrascleral prosthesis implantation, or pharmacologic ciliary body ablation to manage pain and intraocular pressure [1].

The presence of intravitreal silicone oil raises important considerations for intraocular drug delivery, as cidofovir is a water‐soluble agent and its distribution and pharmacologic activity may be altered in the presence of SiO, which displaces the native vitreous and modifies intraocular fluid dynamics [11]. While intravitreal injections of cidofovir have been described in human patients with SiO‐filled eyes, there is a lack of data evaluating its efficacy in dogs under similar conditions [12].

The objective of this case series was to describe the clinical outcome of intravitreal cidofovir injection for end‐stage glaucoma following RRS with SiO tamponade, and to evaluate the IOP‐lowering effect and post‐treatment clinical comfort in these cases.

## 2 Case Description

### 2.1 Case Selection and Clinical Background

Informed consent for treatment and data collection was obtained from all owners. Four client‐owned dogs with vision loss secondary to glaucoma following RRS were included in this case series. All dogs had undergone RRS for rhegmatogenous retinal detachment using pars plana vitrectomy and endolaser retinopexy, followed by SiO tamponade. Two dogs (Cases 3 and 4) additionally underwent concurrent phacoemulsification with intraocular lens (IOL) implantation due to pre‐existing cataracts. Glaucoma was diagnosed between 2 and 14 months after RRS, and all eyes showed progressive vision loss with inadequate IOP control despite medical management.

Given the presence of SiO, owners were counseled regarding the potential for altered distribution and efficacy of an aqueous‐phase drug, as well as the possibility of unanticipated complications. All owners declined enucleation and elected pharmacologic ciliary body ablation using intravitreal cidofovir injection as a palliative alternative. Signalment and baseline clinical findings, including pre‐injection IOP values, are summarized in Table 1.

**TABLE 1:** Clinical characteristics and intraocular pressure outcomes of dogs treated with intravitreal cidofovir injection.

Case	Breed	Age at cidofovir injection (years)	Sex	Onset of glaucoma (post‐retinopexy)	Lens status	Pre‐injection IOP (mmHg)	Post‐injection IOP (days post‐injection)	Follow‐up (days)
Case 1 (OD)	Mixed	13	Male castrated	7 months	Phakic	50	7 (14)	369
Case 2 (OD)	Maltese	13	Spayed female	14 months	Phakic	55	5 (14)	361
Case 3 (OD)	Toy Poodle	8	Spayed female	10 months	Pseudophakic^a^	47	3 (28)	252
Case 4 (OS)	Yorkshire terrier	13	Spayed female	2 months	Pseudophakic^a^	40	6 (19)	40

^a^ Phacoemulsification with intraocular lens implantation was performed concurrently with retinal reattachment surgery.

### 2.2 Intravitreal Cidofovir Injection Protocol

All procedures were performed under topical anesthesia using 0.5% proparacaine hydrochloride (Alcaine; Alcon, Fort Worth, TX, USA) ophthalmic solution applied with cotton‐tipped applicators to the injection site for approximately 1 min. Prior to injection, the eyelid margins and conjunctival fornices were disinfected thoroughly using 0.5% povidone‐iodine solution.

Anterior chamber paracentesis was performed at the limbus using a 30‐gauge needle to remove approximately 0.1 mL of aqueous humor and mildly reduce IOP. Subsequently, 0.12 mL of 0.5% cidofovir (approximately 600 micrograms per globe), prepared from sterile cidofovir powder (Sigma‐Aldrich, St. Louis, MO, USA), was injected intravitreally via a trans‐scleral approach approximately 5 mm posterior to the limbus. The dosage and technique were based on the protocol described by previous study [3]. No immediate adverse effects, such as globe rupture or intraocular hemorrhage, were observed following intravitreal injection. Following injection, all dogs received a subconjunctival injection of dexamethasone sodium phosphate (Dexa‐S; Jeil Pharm. Co. Ltd., Seoul, Republic of Korea) and gentamicin sulfate (Gentamicin; Shin Poong Pharm. Co. Ltd., Seoul, Republic of Korea). Subconjunctival gentamicin was administered prophylactically to prevent bacterial infection, and subconjunctival dexamethasone, a short‐acting corticosteroid, was used to mitigate the anticipated acute inflammatory response associated with intravitreal cidofovir injection. Post‐injection treatment included topical 0.3% ofloxacin (Ocuflox; Samil Pharm. Co. Ltd., Seoul, Republic of Korea) ophthalmic solution, administered three times daily (TID) to four times daily (QID) until the first post‐injection recheck (14–28 days), to minimize the risk of post‐injection infectious complications including endophthalmitis. Sustained anti‐inflammatory control was maintained with topical ophthalmic medications throughout the post‐injection period. Topical anti‐inflammatory drugs were prescribed in all four cases: 1% prednisolone acetate (Pred Forte; Allergan, Irvine, CA, USA), administered two to three times daily (BID to TID), was used in Cases 1, 2, and 4, while 0.1% diclofenac sodium (Optanac; Kukje Pharma Co. Ltd., Seongnam, Republic of Korea), administered twice daily (BID), was used in Case 3 due to elevated hepatic enzyme values.

### 2.3 Clinical Outcome and Follow‐Up

Clinical follow‐up ranged from 40 to 369 days among the four cases (median, 306.5 days). All treated eyes showed a substantial reduction in IOP at the first post‐injection recheck, with IOPs of 7, 5, 3, and 6 mmHg measured at 14, 14, 28, and 19 days after injection in Cases 1–4, respectively. Adequate pain control was achieved in all dogs, and no signs of ocular discomfort were observed during the follow‐up period. At the final examination, progressive phthisis bulbi was noted in Case 1 (369 days post‐injection). Phthisis bulbi was not observed in the remaining three cases during the available follow‐up period (40–361 days); however, Case 3 demonstrated resolution of pre‐existing buphthalmos. Throughout the extended follow‐up period, all cases remained clinically stable with sustained IOP control and no signs of recurrent ocular discomfort.

All eyes developed moderate to severe post‐injection ocular discharge, conjunctival hyperemia, and aqueous flare. These findings were manageable with routine topical therapy, including artificial tears and intermittent topical anti‐inflammatory medications. Overall, owners reported satisfaction with the post‐injection cosmetic appearance of the treated eyes.

## 3 Discussion

The results of this case series demonstrate that intravitreal cidofovir injection can achieve consistent IOP reduction and satisfactory pain control in SiO‐filled eyes with refractory glaucoma. Despite theoretical concerns regarding drug distribution in the presence of hydrophobic SiO, all treated eyes showed marked hypotony following a single injection. These findings align with previous reports in human ophthalmology demonstrating that intravitreal delivery of aqueous‐phase drugs may retain clinical effectiveness in SiO‐filled eyes [12].

In human studies, several aqueous‐phase agents, including cidofovir, anti‐vascular endothelial growth factor (anti‐VEGF) agents, and corticosteroids, have shown measurable clinical effects following intravitreal administration in SiO‐filled eyes [12, 13]. Rahhal et al. reported successful treatment of cytomegalovirus retinitis using intravitreal cidofovir in SiO‐filled eyes, directly observing a distinct aqueous drug bubble forming within the oil‐filled vitreous cavity. The therapeutic effect was achieved without dissolution of the drug into the silicone oil itself, supporting the concept that effective drug activity may occur within a segregated aqueous compartment [12]. Similarly, Safadi et al. described partial but reproducible responses to intravitreal anti‐VEGF and steroid injections in SiO‐filled eyes with macular edema [13]. These findings challenge the assumption that silicone oil completely precludes aqueous drug efficacy.

The prevailing explanation is that SiO does not entirely replace the aqueous component of the vitreous cavity. A residual aqueous layer—often localized inferiorly beneath the SiO bubble—remains present, allowing aqueous‐phase drugs to persist and diffuse toward their target tissues. Gravity‐driven sedimentation and slow diffusion within this compartment may enable sufficient drug exposure over time, even without homogeneous distribution throughout the vitreous cavity [12, 14].

A similar mechanism may apply to the present canine cases. The target tissue in pharmacologic ciliary body ablation differs from retinal targets described in human studies [12, 13]. The ciliary body epithelium is continuously bathed in aqueous humor and may therefore be more accessible to drugs diffusing within the aqueous phase, even when SiO occupies the vitreous cavity. Following injection, cidofovir likely remained in the inferior aqueous compartment beneath the SiO and gradually diffused toward the ciliary epithelium, leading to effective ablation and reduced aqueous production. This hypothesis is consistent with the rapid IOP reduction and sustained pain control observed in all cases. Given the palliative intent, this indirect diffusion mechanism appears clinically sufficient.

Regarding the development of phthisis bulbi in Case 1, phthisis bulbi has been reported in 42.5%–70% of cidofovir‐treated eyes and is attributed to cidofovir‐induced cytotoxic destruction of the ciliary body leading to sustained hypotony [3, 4, 5]. In blind, painful eyes, such structural changes do not necessarily indicate treatment failure, particularly when pain control and acceptable cosmesis are maintained. Although phthisis bulbi was not observed in the remaining three cases during the available follow‐up period (40–361 days), the possibility of its subsequent development could not be excluded given the limited follow‐up durations.

Several limitations should be acknowledged. First, the proposed mechanism of drug distribution in SiO‐filled eyes remains inferential, as direct visualization of intraocular cidofovir distribution was not performed. Second, the small number of cases limits generalizability, and although follow‐up extended up to 369 days, longer observation is needed to fully characterize late complications. Whether late complications differ from those in eyes without SiO tamponade requires further investigation.

Despite these limitations, the present case series suggests that intravitreal cidofovir injection may serve as a feasible palliative option for managing refractory glaucoma in SiO‐filled eyes when enucleation is declined. This approach may provide pain relief and IOP control in carefully selected cases. Given that different agents may exhibit distinct diffusion characteristics in oil‐filled eyes, further studies with larger cohorts and longer follow‐up periods, including comparisons of alternative drugs such as gentamicin, are warranted to refine treatment protocols and better define long‐term outcomes in this clinical setting.

## 4 Conclusion

In conclusion, a single intravitreal cidofovir injection achieved consistent IOP reduction and pain control in SiO‐filled eyes with refractory glaucoma in this case series, supporting its potential as a palliative alternative to enucleation. Further studies are warranted to clarify long‐term safety and outcomes in this clinical setting.

## Author Contributions

**Sunhyo Kim:** conceptualization, investigation, writing – original draft. **Jaeho Shim:** writing – review and editing, visualization, supervision, data curation.

## Funding

This work (GNU‐2024‐240611) was supported by the research grant of the new professor of the Gyeongsang National University in 2024.

## Disclosure

The authors have not used AI to generate any part of the manuscript.

## Ethics Statement

This study was a retrospective review of clinical records and did not require formal Institutional Animal Care and Use Committee (IACUC) approval. All procedures were performed as part of routine clinical cases. Written informed client consent was obtained from the owners of all animals included in this study.

## Conflicts of Interest

The authors declare no conflicts of interest.

## Data Availability

The data that support the findings of this study are available from the corresponding author upon reasonable request.
